# Intravenous Lidocaine versus Morphine Sulfate in Pain Management for Extremity Fractures; a Clinical Trial

**Published:** 2017-05-28

**Authors:** Arash Forouzan, Hassan Barzegari, Hassan Motamed, Ali Khavanin, Hamideh Shiri

**Affiliations:** 1 Emergency Medicine Department, Ahvaz Jundishapur University of Medical Sciences, Ahvaz, Iran.

**Keywords:** Lidocaine, morphine, pain management, emergency service, hospital, fractures, bone

## Abstract

**Introduction:**

Considering the existing contradictions regarding effectiveness of intravenous (IV) lidocaine, especially in emergency department (ED), the present study was designed to compare the analgesic effect of IV lidocaine and morphine sulfate in pain management for extremity bone fractures.

**Method:**

In this triple blind clinical trial, 15 to 65 year-old patients with extremity fractures and in need of pain management were randomly allocated to either IV lidocaine or morphine sulfate group and were compared regarding severity of pain 5, 10, 15, 20, 25, and 30 minutes after infusion via intention to treat analysis. The absolute risk reduction, number needed to treat and relative risk of IV lidocaine after 30 minutes were 0.40 (95%CI: 0.25 – 0.64), 7 (95%CI: 3.7 – 23.1), and 20.71 (95%CI: 10.91 – 30.51), respectively.

**Results:**

280 patients with the mean age of 32.50 ± 12.77 years were randomly divided into 2 equal groups of 140 (73.9% male). The 2 groups had similar baseline characteristics. 15 minutes after injection success rate was 49.28% in lidocaine and 33.57% in morphine sulfate group (p = 0.011), and after 30 minutes it reached 85.71% and 65.00%, respectively (p < 0.001).

**Conclusion:**

Based on the results of the present study, IV lidocaine could be considered as a reasonable alternative choice for pain management in ED.

## Introduction

Pain is an unpleasant mental and sensory experience that often occurs after a tissue injury and secretion of inflammatory cytokines (1). Various methods, such as using opioids and non-steroid anti-inflammatory drugs, topical anesthetics and regional nerve block are applied for pain management (2-6). Intravenous (IV) infusion of lidocaine is one of the methods used by anesthesiology specialists for induction of analgesia (7-11). Lidocaine is a relatively safe drug in the amide group, which acts as an analgesic, anti-hyperalgesia and anti-inflammatory agent in low doses and is affective in relieving neuralgia, burn and procedural pains (12). This drug induces its analgesic effects via stimulating secretion of anti-inflammatory cytokines (interleukin-1) receptor antagonist and blocking central and peripheral voltage-dependent sodium channels (13). In cases that opioids lack efficient effectiveness, IV injection of lidocaine has been used as a proper replacement (12, 14, 15). 

Although many studies have indicated the role of IV lidocaine in pain relief after trauma or surgery and decrease in the need for other opioids, there are also studies that do not agree (7). For example, in one study continuous infusion of low doses of lidocaine, had not reduced use of other analgesics (16). In addition, for induction of analgesia after tonsillectomy surgery, infusion of IV lidocaine did not play an effective role in reducing pain after surgery (17).

Therefore, considering the limited number of studies on the effect of IV lidocaine in pain management, especially in emergency department (ED) and the existing contradictions regarding its effectiveness, the present study was designed with the aim of assessing the analgesic effect of IV lidocaine compared to IV morphine sulfate in relieving the pain caused by traumatic extremity bone fractures.

## Methods

***Study design***

The present clinical trial was carried out during a 6 month period from June 2016 to Nov 2016 on patients presenting to ED of Imam Khomeini and Golestan Hospitals in Ahvaz, Iran, who needed pain management due to traumatic bone fractures. Before including the patients in the study, informed consent was obtained from them and they were given explanations regarding the study process. Throughout all steps of the study, the researchers adhered to the principles of Helsinki Declaration and confidentiality of patient data. All costs of the project were covered by the researchers and no additional costs were inflicted on the patients. This study was approved by the ethics committee of Ahvaz University of medical sciences and has been registered on Iranian registry of clinical trials under the number IRCT2015052622423N1.

***Participants***

The study population consisted of patients aged 15 to 65 years who had fractures in the long bones of their upper or lower extremities and the fracture was evident in physical examination and the patient needed pain management (moderate to severe pain). Pregnant and lactating women; patients with altered level of consciousness; hemodynamic instability; evidence of intra-abdominal or pelvic hemorrhage; mental retardation; history of allergy to lidocaine or morphine; history of addiction; underlying illness (coronary artery problems, valvular disorders, arrhythmia, hypertension, cerebrovascular diseases, seizure, diabetes, liver or kidney diseases); and consuming cardiac drugs were excluded from the study. No limitations were considered regarding sex and fracture type and being open or closed fracture. Physical examination and patient selection was done by a senior emergency medicine resident and a senior orthopedics resident.

***Intervention***

After selecting the patients that met the inclusion criteria of the study, they were divided into 2 groups receiving either IV lidocaine (1.5 mg/kg during 2 minutes) or IV morphine sulfate (0.1 mg/kg during 2 minutes). The study was designed as a triple blind manner as the patient, the person injecting the drug, and data analyzer were all blind to the type of drug consumed. Both drugs were colorless and odorless and to make them look alike, both drugs were injected in a 10cc volume with syringes with the same shape and color. Injection was performed by either an emergency medicine specialist or a senior resident of emergency medicine under complete cardiac, respiratory, blood pressure, level of consciousness and pulse oximetry monitoring. Before the injection of drug, vital signs of the patient and their pain score using visual analog scale (VAS) were measured and recorded.

***Data gathering***

A checklist consisting of demographic data (age, sex), vital signs (number of breaths per minute, systolic and diastolic blood pressures, heart beats per minute, and oxygen saturation percentage) and pain severity on presentation and 5, 10, 15, 20, 25, and 30 minutes after injection was filled for all the patients. The senior emergency medicine resident in charge of the patient was responsible for gathering the data, but evaluation of vital signs of the patient was performed by someone other than the one injecting the drugs, who was blind to the type of drug used.

To measure pain severity, VAS scale was used. Pain score of 3 to 6 was considered as moderate pain and score ≥6 was considered as severe pain. At least 3 points drop in pain score was considered as success in pain management.

If pain was still present after 30 minutes, IV fentanyl with 1.5 mg/kg dose was prescribed for the patients as bolus.

***Outcome***

At least 3 scores drop in pain severity was considered as success and less than 3 scores as failure in treatment on 15^th^ and 30^th^ minutes. In addition, patients were assessed regarding manifestation of any side effects such as confusion; tremor; stupor; seizure; restlessness; anxiety; lethargy; sleepiness; hallucination; strabismus; syncope; hypotension; bradycardia; cardiac failure; new arrhythmia; cardiac failure; anaphylaxis; status asthmaticus; edema; nausea; vomiting; rash and tinnitus. It was determined that in case of any drug side effects, the patient should be excluded from the study and be rapidly treated for relieving the side effect.

***Statistical analysis***

Non-probability convenience sampling was used and analysis of data was done with intention to treat method via SPSS 21 statistical software. Quantitative variables were reported as mean ± standard deviation (SD), and qualitative data as frequency and percentage. To report the effectiveness of the drug, absolute risk reduction (ARR), number needed to treat (NNT) and relative risk (RR) were used with 95% CI. To do comparisons between the 2 groups, chi square and t-test were used. P < 0.05 was considered as level of significance.

## Results

**Figure 1:**
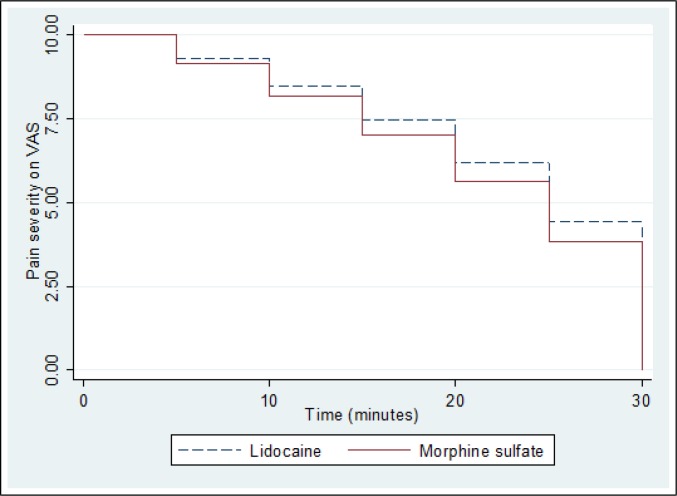
Changes in pain severity measures in the 2 studied groups at different times after intravenous injection of the drug

280 patients with the mean age of 32.50 ± 12.77 years (15 - 65) were randomly divided into 2 equal groups receiving either IV lidocaine (140 patients) or morphine sulfate (140 patients) (73.9% male). Table 1 compares baseline data of the participants in the 2 groups. The 2 groups had similar characteristics regarding pain severity and vital signs on admission to ED. Table 2 and figure 1 depict the rate of decrease in pain severity in the 2 studied groups at various times after IV injection. Success rate in decreasing pain severity (at least 3 points) 15 minutes after injection was 49.28% (69 individuals) in lidocaine group and 33.57% (47 patients) in morphine sulfate group (p = 0.011), and 30 minutes after injection, these numbers were 85.71% (120 patients) and 65.00% (91 individuals), respectively (p < 0.001).

No cases of hypotension, respiratory depression, dysrhythmia and drop in arterial oxygen saturation were detected in either group during the initial 30 minutes after drug injection.

**Table 1:** Comparison of baseline data of the patients in the 2 study groups on admission to emergency department

Variable	**Groups**		P value
	**IV** * ** lidocaine**	**IV** * ** morphine**	
**Age (year)**	31.47 ± 12.31	33.53 ± 13.16	0.178
**Sex **			
Male	104 (50.2)	103 (49.8)	0.500
Female	36 (49.3)	37 (50.7)	
**Pain severity**			
Moderate	12 (8.6)	17 (12.1)	0.434
Severe	128 (91.4)	123 (87.9)	
**Heart rate (per minute)**	89.06 ± 6.98	88.19 ± 8.98	0.328
**Respiratory rate (per minute)**	17.93 ± 1.69	18.30 ± 1.81	0.078
**Systolic blood pressure (mmHg)**	123.85 ± 11.68	124.5 ± 13.46	0.667
**Diastolic blood pressure (mmHg)**	76.21 ± 6.90	77.26 ± 9.09	0.281
**Oxygen saturation (%)**	98.46 ± 0.76	98.36 ± 0.71	0.293

* IV: intravenous; Data are presented as frequency and percentage or mean ± standard deviation. Pain severity is shown based on VAS score.

**Table 2:** Comparing the pain severity of the patients between the 2 studied groups in the initial 30 minutes after intravenous (IV) injection of the drug

**Pain severity**	**Study groups; no (%)**		**P value**
	**IV lidocaine **	**IV morphine **	
**15 minutes**			
Mild	10 (7.1)	10 (7.1)	0.884
Moderate	70 (50.0)	66 (47.1)	
Severe	60 (42.9)	64 (45.8)	
**30 minutes**			
Mild	51 (36.4)	37 (27.1)	0.186
Moderate	58 (41.5)	61 (43.6)	
Severe	31 (22.1)	41 (29.3)	

**Table 3:** Effectiveness characteristics of intravenous lidocaine in pain management of patients with extremity fractures

Characteristics	**Values (95% CI)**	
	**After 15 minutes**	**After 30 minutes**
**MS failure rate **	66.43%	35.00%
**Lidocaine failure rate **	50.71%	14.29%
**Relative risk**	0.76 (0.62 – 0.93)	0.40 (0.25 – 0.64)
**Absolute risk reduction**	15.72 (4.33 – 27.11)	20.71 (10.91 – 30.51)
**Number needed to treat**	5 (3.3 – 9.2)	7 (3.7 – 23.1)

MS: morphine sulfate

## Discussion

Based on the results of the present study, success rate of IV lidocaine in relieving the pain caused by extremity fractures was significantly higher than that of morphine sulfate, 15 and 30 minutes after injection. Other indices of effectiveness such as RR, ARR and NNT also confirmed the proper effect of IV lidocaine for management of acute pain. In addition, no drug side effect was detected in either group.

While about half of the patients who received IV lidocaine experienced at least 3 points drop in their pain score during the first 15 minutes after injection, this rate was 33% for the group receiving IV morphine. In other words, using lidocaine led to 15% to 20% drop in unsuccessful pain management cases 15 and 30 minutes after injection.

Relative risk or risk ratio of using lidocaine 15 and 30 minutes after injection were 0.40 and 0.76, respectively. In other words, the probability of not controlling the pain in lidocaine group relative to morphine sulfate group was less than 1, which confirms its higher effectiveness.

Numerous studies have confirmed the effectiveness of IV lidocaine for successful pain management and its role in decreasing the need for opioid use (12, 14, 15). Low dose of IV lidocaine has been safer and more effective than regional analgesia in relieving pain caused by forearm fracture in children (18). 

A clinical trial by El-Tahan et al. in 2009 showed that IV lidocaine prescription before cesarean section surgery leads to less increase in heart rate, average arterial pressure and concentration of plasma cortisol. Their findings indicated that IV lidocaine can be considered as a safe and effective choice for reducing stress response of the mother to surgery during cesarean delivery (19).

Findings of Yardeni et al. in 2009 showed that injection of IV lidocaine before and during surgery can be effective in improvement of pain management after surgery as well as reduction of immune interference and release of pain and inflammation inducing mediators due to surgery (20).

Need for morphine sulfate in the first 72 hours post operation was lower in the group that had received IV lidocaine before surgery (21).

Based on the findings of the present study, it seems that IV lidocaine has proper effectiveness in controlling acute pain caused by extremity fractures. Probably, the main reason for the low rate of using this option for pain management in ED is fear of side effects such as dysrhythmia. It seems that since most of the patients participating in the present study were either young or in their midlife, the probability of showing cardiac side effects was naturally lower. It seems more logical to decide on the side effects of injecting the drug after studies on various age groups and considering their various underlying illnesses.

If the results of the present study are confirmed in other studies, we can count on IV lidocaine as an option and proper replacement for pain management of patients in ED.

Limitations

Longer follow up of the patients and calculating the patients’ need for using analgesics after probable relapses are among the points that can decrease the limitations of the present study. Measuring serum level of lidocaine to avoid reaching toxic levels of the drug can also be considered in future studies. Repeating the study in populations with various age ranges and considering various types of underlying illnesses can be beneficial. In addition, for a more accurate comparison, fracture types and their location should better be homogenized between the 2 groups. However, random allocation of the patients prevented this confounding factor affecting the results to a great extent in the current study.

## Conclusions

Based on the results of the present study, success rate of IV lidocaine in relieving pain caused by extremity fracture was significantly higher than IV morphine. Using lidocaine led to 20% decrease in unsuccessful pain management cases until 30 minutes after injection with risk ratio of 0.76.
